# Erratum

**Published:** 1867-08

**Authors:** 


### *Erratum.*

In the advertisement by Messrs. Bliss and Sharp, 144 Lake street, on the cover of the Announcement of Rush College, by typographical error the name *Crenshaw* is made *Ciershaw.* The name of the celebrated pharmaceutic firm, BULLOCK & CRENSHAW, is too well known to require correction of this manifest error, but we make it in deference to the request of Messrs. Bliss & Sharp.

